# Electrochemical synthesis of nitric acid from air and ammonia through waste utilization

**DOI:** 10.1093/nsr/nwz019

**Published:** 2019-02-01

**Authors:** Yuting Wang, Yifu Yu, Ranran Jia, Chao Zhang, Bin Zhang

**Affiliations:** 1 Department of Chemistry, Institute of Molecular Plus, School of Science, Tianjin University, Tianjin 300072, China; 2 Collaborative Innovation Center of Chemical Science and Engineering, Tianjin 300072, China

**Keywords:** ammonia, nitric acid, distributed sources, electrocatalysis, green synthesis

## Abstract

Commercial nitric acid (HNO_3_) and ammonia (NH_3_) are mostly produced through the Ostwald process and the Haber-Bosch process, respectively. However, high energy demand and enormous greenhouse gas accompy these processes. The development of economical and green ways to synthesize HNO_3_ and NH_3_ is highly desirable for solving the global energy and environmental crisis. Here, we present two energy-efficient and environmentally friendly strategies to synthesize HNO_3_ and NH_3_ at distributed sources, including the electrocatalytic oxidation of N_2_ in air to HNO_3_ and the electrocatalytic reduction of residual ${\rm NO_{3}^{-}}$ contamination in water to NH_3_. The isotope-labeling studies combined with theoretical calculation reveal the reaction path of the two proposed strategies, confirming the origin of the electrochemical products. Importantly, the electrooxidation-generated ${\rm NO_{3}^{-}}$ ions may also serve as reactants for the electroreduction synthesis of NH_3_ in the future. Our work may open avenues for energy-efficient and green production of HNO_3_ and NH_3_ at distributed sources.

## INTRODUCTION

Transformations of nitrogen into reactive forms, particularly nitric acid (HNO_3_) and ammonia (NH_3_), are vital to living organisms and industrial processes [1–4]. The worldwide production of HNO_3_ and NH_3_ was 50 and 150 million metric tons, respectively, in 2017 [5]. Commercial HNO_3_ is produced through the catalytic oxidation of NH_3_ (Ostwald process), but this approach is energy-intensive [5]. On the other hand, NH_3_ is mostly manufactured using the Haber-Bosch method [6–9], which consumes ∼2% of global power and discharges ∼1.5% of global greenhouse gas [10–16]. Moreover, the large-scale chemical plants, and hence centralized HNO_3_ and NH_3_ sources, induce a serious waste of fossil fuels during the transportation process [17]. Therefore, searching novel solutions that allow energy-efficient and environmentally friendly synthesis of HNO_3_ and NH_3_ at distributed sources are urgently needed [18,19]. Electrocatalysis represents a potentially alternative strategy [20]. However, present electrocatalysis is focused on preparing NH_3_ through the reduction of pure dinitrogen, which is produced by the energy-intensive air-separation process [21–24]. Thus, it is of great interest to develop novel electrochemical routes to achieving green synthesis of HNO_3_ and NH_3_ under benign conditions.

Herein, we present two electrochemical strategies. Strategy I is the electrocatalytic oxidation of N_2_ to HNO_3_ by using air as the nitrogen source. Strategy II is the electrochemical reduction preparation of NH_3(aq)_ from residual nitrate ion (${\rm NO_{3}^{-}}$) contamination in water [25,26]:

(1) \begin{equation*} {\rm{NO}}_{3\;({\rm{aq}})}^ - \!+\! 6{{\rm{H}}_2}{{\rm{O}}_{({\rm{liq}})}} + 8{{\rm{e}}^ - }\! \to \!{\rm{N}}{{\rm{H}}_{3({\rm{aq}})}} \!+\! 9{\rm{OH}}_{({\rm{aq}})}^ - . \end{equation*}

We found that N_2_ is electro-oxidized into HNO_3_ over platinum foil with ∼1.23% Faradaic efficiency at +2.19 V vs. RHE (the reversible hydrogen electrode) and the waste of ${\rm NO_{3}^{-}}$ is electro-reduced with approximately 33.6% NH_3(aq)_ selectivity at −0.65 V vs. RHE over Co_3_O_4_ nanorod arrays. Our results demonstrate how the electrochemical methods of Strategy I and Strategy II produce HNO_3_ and NH_3_ at distributed sources. These findings provide a new avenue for producing the reactive nitrogen species in an ‘economic’ and ‘clean’ way, especially once the electrocatalysis reaction is driven by renewable energy [27].

## RESULTS AND DISCUSSION

**Figure 1.**
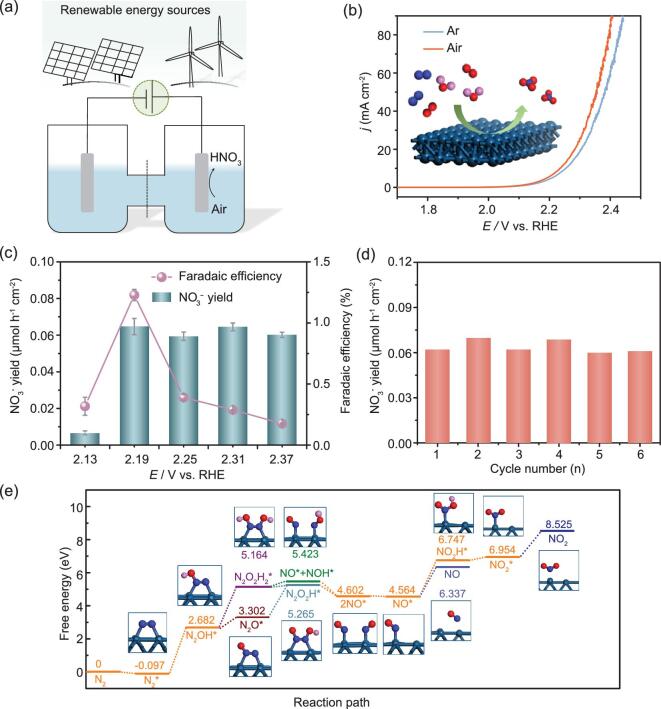
(a) Schematic illustration for as-proposed Strategy I. (b) Linear sweep voltammetric curves of platinum foil in Ar-saturated (blue line) and air-saturated (red line) electrolyte. (c) Yields of ${\rm NO_{3}^{-}}$ and Faradaic efficiencies after potentiostatic test at given potentials. (d) The cycling tests of N_2_ electrooxidation over the same piece of Pt electrode. (e) Calculated free-energy diagram for N_2_ electrooxidation over platinum foil. Ball-and-stick model in (b) and (e): blue ball (N atom), red ball (O atom), pink ball (H atom) and green ball (Pt atom).

An H-type cell divided by a proton-exchange membrane was used for the electrocatalytic tests of Strategy I (Fig. 1a). To explore the catalytic behavior of N_2_ electrooxidation (Strategy I), air was bubbled onto the anode. H_2_O in electrolyte (0.3 M K_2_SO_4_) and N_2_ in air combine with platinum foil as an electrocatalyst to form HNO_3_. We tested the linear sweep voltammetry curves of platinum foil in Ar- and air-saturated electrolyte under ambient conditions (Fig. 1b). All potentials in this work were recorded and converted to the RHE scale. As the potential moves above +2.13 V, the current density is distinguishably enhanced under air-saturated electrolyte, revealing that N_2_ in air can be catalysed into oxidative products. The produced ${\rm NO_{3}^{-}}$ and ${\rm NO_{2}^{-}}$ are quantified based on the standard method [28] by using ultraviolet-visible (UV-Vis) spectrophotometry (Supplementary Fig. S1, available as Supplementary Data at *NSR* online). Anion chromatography was also adopted to confirm the accuracy of UV-Vis spectrophotometry for detecting the yield of ${\rm NO_{3}^{-}}$ (Supplementary Table S1, available as Supplementary Data at *NSR* online). The effect of the anode potentials on the yields of oxidative products and the corresponding Faradaic efficiencies were investigated (Fig. 1c). Although the gap in the current density between Ar and air condition keeps enlarging with the increase in potential, the highest Faradaic efficiency of 1.23% for ${\rm NO_{3}^{-}}$ was obtained at +2.19 V. The yields of ${\rm NO_{3}^{-}}$ and ${\rm NO_{2}^{-}}$ at +2.19 V achieve 0.06 and 0.0004 μmol h^−1^ cm^−2^, respectively, and remained almost unchanged with the increase in potential (Fig. 1c and Supplementary Fig. S2, available as Supplementary Data at *NSR* online). So, the optimal operating potential for N_2_ electrooxidation over platinum electrocatalyst in this work was +2.19 V. Furthermore, some control experiments, including Ar-saturated electrolyte with a potentiostatic test (+2.19 V) and air-saturated electrolyte without external potential, were performed. Undetected oxidative products in both cases further confirmed the electrocatalytic oxidation of N_2_ in air to ${\rm NO_{3}^{-}}$ and ${\rm NO_{2}^{-}}$ as designed in Strategy I (Supplementary Table S2, available as Supplementary Data at *NSR* online). For practical application, the durability of the catalyst is crucial. After consecutive recycling tests, the catalytic performance showed no obvious decline, demonstrating the high stability of platinum foil toward the electrooxidation of N_2_ (Fig. 1d). As shown in Fig. 1e, Strategy I goes through multiple processes. We calculated the free energy of N_2_ electrooxidation over the Pt (200) plane based on the X-ray diffraction (XRD) result (Supplementary Fig. S3, available as Supplementary Data at *NSR* online). First, the N_2_ molecule was chemically absorbed by the platinum to form N_2_* with a total energy change of −0.097 eV, indicating that this reaction can take place spontaneously. N_2_* reacted with OH^−^ to produce N_2_OH* and then N_2_OH* was dehydrogenated to N_2_O* or continued to react with OH^−^ to produce N_2_O_2_H_2_* with a larger reaction energy. Both N_2_O* and N_2_O_2_H_2_* will evolve into NO* with the intermediate of N_2_O_2_H* and NOH*. Note that NO* could be directly desorbed from the catalyst surface and then oxidized into HNO_3_ and HNO_2_ in solution (Equation (2)). Meanwhile, NO* can be oxidized to NO_2_* with the intermediate of NO_2_H*. With further increase in potential, NO_2_* desorbed from the catalyst surface. Finally, NO_2_ was transformed to HNO_3_ in solution through Equation (3). Based on these results, we can deduce the reaction path from N_2_ in air to HNO_3_ via the as-proposed Strategy I:

(2) \begin{eqnarray*} 2{\rm{N}}{{\rm{O}}_{({\rm{g}})}} + {{\rm{H}}_2}{{\rm{O}}_{({\rm{liq}})}} &+& {{\rm{O}}_{2({\rm{g}})}} \to {\rm{HN}}{{\rm{O}}_{3({\rm{aq}})}}\nonumber\\ &+& {\rm{HN}}{{\rm{O}}_{2({\rm{aq}})}}\end{eqnarray*}

(3) \begin{equation*}2{\rm{N}}{{\rm{O}}_{2({\rm{g}})}} + {{\rm{H}}_2}{{\rm{O}}_{({\rm{liq}})}} + 1/2{{\rm{O}}_{2({\rm{g}})}} \to 2{\rm{HN}}{{\rm{O}}_{3({\rm{aq}})}}.\end{equation*}

**Figure 2.**
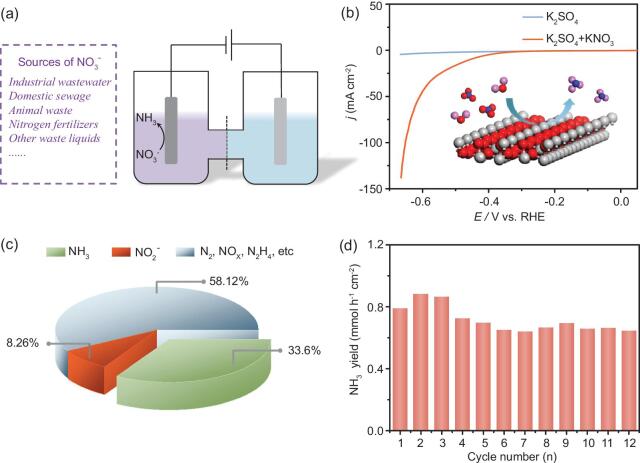
(a) Schematic illustration for as-proposed Strategy II. (b) Linear sweep voltammetric curves of Co_3_O_4_ electrode in 0.1 M K_2_SO_4_ electrolyte with and without KNO_3_. (c) Selectivity of ${\rm NO_{3}^{-}}$ electroreduction products at −0.65 V vs. RHE. (d) The cycling tests of ${\rm NO_{3}^{-}}$ reduction over the same piece of Co_3_O_4_ electrode.

To demonstrate Strategy II, involving electroreducing residual ${\rm NO_{3}^{-}}$ contamination in water, KNO_3_ was added to a cathode cell and reduced into NH_3(aq)_ (Fig. 2a). Presently, the main sources of nitrate for the as-proposed Strategy II are residual contamination in water, including industrial wastewater, domestic sewage, animal waste and nitrogen fertilizers. In the future, if the efficiency of Strategy I can be further improved, the electrooxidation generation of ${\rm NO_{3}^{-}}$ may also serve as a reactant for Strategy II. We constructed Co_3_O_4_ nanorod arrays supported on Ti mesh as a model electrocatalyst (Supplementary Fig. S4, available as Supplementary Data at *NSR* online). The linear sweep voltammetry curves of the Co_3_O_4_ electrode in 0.1 M K_2_SO_4_ electrolyte with and without KNO_3_ were performed under ambient conditions (Fig. 2b). The current density was obviously enhanced in the presence of KNO_3_, indicating that ${\rm NO_{3}^{-}}$ in solution can be catalysed into reductive products. The yields of NH_3(aq)_ [28] and ${\rm NO_{2}^{-}}$ were quantified based on UV-Vis spectrophotometers (Supplementary Fig. S5, available as Supplementary Data at *NSR* online). The cation chromatography method was also adopted to confirm the accuracy of UV-Vis spectrophotometry for detecting the yield of NH_3(aq)_ (Supplementary Table S3, available as Supplementary Data at *NSR* online). Following the potentiostatic test conducted at −0.65 V vs. RHE, which corresponded to 100 mA cm^−2^ of current density (Fig. 2b), the selectivity of different reductive products is displayed in Fig. 2c. The selectivity of NH_3(aq)_ was 33.6% over the Co_3_O_4_ electrode, and the other reductive products included ${\rm NO_{2}^{-}}$, NO_x_, N_2_ and N_2_H_4_, etc. [29–31]. Furthermore, the Co_3_O_4_ electrode supported on the Ti substrate was conducted under a potentiostatic test (–0.65 V) in 0.1 M K_2_SO_4_ electrolyte without KNO_3_. The negligible NH_3(aq)_ in the final solution further confirms the electrocatalytic reduction of ${\rm NO_{3}^{-}}$ to NH_3(aq)_ via Strategy II (Supplementary Fig. S6, available as Supplementary Data at *NSR* online). The bare Ti substrate showed a much smaller yield of NH_3(aq)_ (0.029 mmol h^−1^ cm^−2^) compared to that of Co_3_O_4_ supported on the Ti substrate (0.854 mmol h^−1^ cm^−2^), indicating the high activity of Co_3_O_4_ for the electrocatalytic reduction of ${\rm NO_{3}^{-}}$ (Supplementary Fig. S6, available as Supplementary Data at *NSR* online). During the consecutive recycling test, the catalytic activities displayed almost no decline (Fig. 2d). And the Co_3_O_4_ electrode was maintained well after the electroreduction test (Supplementary Fig. S7, available as Supplementary Data at *NSR* online). These results demonstrate the high durability of the Co_3_O_4_ electrode for ${\rm NO_{3}^{-}}$ electroreduction. Note that the electroreduction of ${\rm NO_{3}^{-}}$ was reported in previous work, but they focused on the degradation of residual ${\rm NO_{3}^{-}}$ in water into environmentally friendly products [32–34]. We herein propose utilization of the waste of ${\rm NO_{3}^{-}}$, provided by environmental contaminations, to produce the high value-added NH_3(aq)_ via electroreduction.

**Figure 3.**
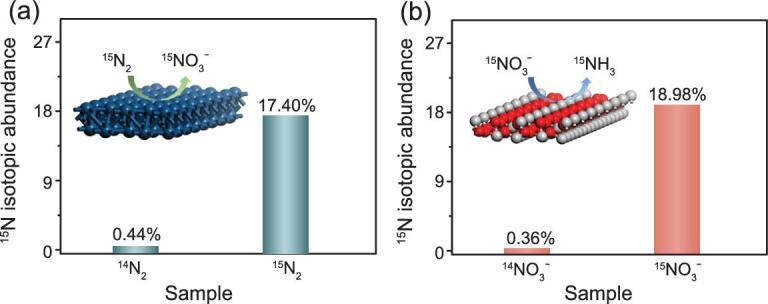
(a) The isotopic mass spectrometry of ^15^${\rm NO_{3}^{-}}$ with ^15^N_2_ and ^14^N_2_ as the feeding gas for Strategy I. (b) The isotopic mass spectrometry of ^15^NH_3(aq)_ with ^15^KNO_3_ and ^14^KNO_3_ as the reactants for Strategy II.

To confirm the origin of the ${\rm NO_{3}^{-}}$ generated from N_2_ electrooxidation, we designed an isotopic-labeling study using ^15^N_2_ (>99 atom% ^15^N) and ^14^N_2_ (with the natural abundance of 0.36 atom% ^15^N) as the feeding gas for Strategy I [35]. As seen in Fig. 3a, the sample using ^14^N_2_ as the feeding gas produced 0.44% ^15^${\rm NO_{3}^{-}}$, while the isotopic-labeled sample showed 17.40% abundance of ^15^${\rm NO_{3}^{-}}$—much higher than the natural abundance of ^15^N. The concentration difference of ^15^N for the isotopic-labeled sample between the ^15^N_2_ reactant and the ^15^${\rm NO_{3}^{-}}$ product may arise from the leaking and/or residue of air in the reactor. These results clearly confirm that the N element of the generated ${\rm NO_{3}^{-}}$ via Strategy I came from N_2_. We also performed an isotopic-labeling study using K^15^NO_3_ (20.3 atom% ^15^N) and K^14^NO_3_ (0.36 atom% ^15^N) as the reactants to explore the origin of the NH_3(aq)_. It can be seen that the isotopic-labeled sample exhibited 18.98% ^15^NH_3(aq)_ but the sample using K^14^NO_3_ without an isotopic label as a reference showed only 0.36% ^15^NH_3(aq)_ (Fig. 3b). These results demonstrate that the N element of the formed NH_3(aq)_ via Strategy II originated from the ${\rm NO_{3}^{-}}$ species.

## CONCLUSIONS

In summary, we present two energy-efficient and environmentally friendly strategies to prepare HNO_3_ and NH_3_ at distributed sources. Strategy I is the electrocatalytic oxidation of N_2_ in air to HNO_3_ and platinum foil is adopted as the model catalyst, showing a generation rate of 0.06 μmol h^−1^ cm^−2^ for HNO_3_ at +2.19 V. Strategy II is the electrocatalytic reduction of residual ${\rm NO_{3}^{-}}$ contamination in water to NH_3(aq)_ and Co_3_O_4_ nanorod arrays supported on a Ti mesh, exhibiting a selectivity of 33.6% for NH_3(aq)_ at −0.65 V. Combined with the theoretical calculation, the reaction path from N_2_ in air to HNO_3_ via the as-proposed Strategy I was deduced. The isotope-labeling studies using ^15^N_2_ and K^15^NO_3_ confirmed the origin of the electrochemical products. Moreover, the platinum electrode and Co_3_O_4_ electrode showed high durability for the electrocatalytic oxidation of N_2_ and the electrocatalytic reduction of ${\rm NO_{3}^{-}}$, respectively. In the future, ${\rm NO_{3}^{-}}$ from Strategy I may also serve as a reactant for the electroreduction synthesis of NH_3_. Our results presented here provide new avenues for energy-efficient and green production of HNO_3_ and NH_3_ at distributed sources.

## METHODS

### Preparation of Co_3_O_4_ cathode for ${\bf NO_{3}^{-}}$ reduction via Strategy II

In a typical process, 2 mmol Co(NO_3_)_2_·6H_2_O, 10 mmol urea and 8 mmol NH_4_F were dissolved in 36 mL distilled water under stirring for 5 min. The aqueous solution was moved to a 50-mL Teflon-lined autoclave and then a piece of Ti mesh (1 × 3 cm^2^) was immersed in the above solution. The autoclave was sealed and heated at 120°C for 9 h, followed by cooling down to ambient temperature. The sample was washed using distilled water and ethanol six times and then dried in a vacuum oven overnight. The dried sample was annealed at 300°C for 2 h in air to acquire the final product of Co_3_O_4_ nanorod arrays supported on a Ti mesh.

### Characterization

The scanning electron microscopy images were acquired from a Hitachi S-4800 scanning electron microscope. Transmission electron microscopy and high-resolution transmission electron microscopy images were taken using a JEOL-2100F system. The XRD was measured using a Bruker D8 Focus Diffraction System with a Cu Kα source (λ = 0.154178 nm). X-ray photoelectron spectrum analysis was recorded via a PHI 5000 Versaprobe system using monochromatic

Al Kα radiation. All binding energies were revised according to the C 1-s peak at 284.8 eV. The ultraviolet-visible (UV-Vis) absorbance spectra were measured on a Beijing Purkinje General T6 new century spectrophotometer. Anion chromatography was performed on an ICS-1100, Thermo. Cation chromatography was conducted on an ICS-900, Thermo. The concentration of ^15^N isotope labeling was established by isotopic mass spectrometry (MAT-271). The pH values of the electrolytes were determined using a pH-meter (LE438 pH electrode, Mettler Toledo, USA).

### Electrochemical measurements

Electrochemical measurements were conducted using an electrochemical workstation (CHI 660D, Chenhua, Shanghai). A typical H-type electrolytic cell divided by a proton-exchange membrane (Nafion 117) was used. Except for special instructions, all potentials were recorded against the RHE. The potentials against the saturated calomel electrode (SCE) were translated to those against the RHE using the following equation: *E* (vs. RHE) = *E* (vs. SCE) + 0.2415 + 0.059 × pH. All the polarization curves were the steady lines after many cycles and the current density was normalized to the geometric surface area.

For the electrooxidation of N_2_ to HNO_3_ via Strategy I, Pt plates (1 × 1 cm^2^) were used as both the working electrode and the counter electrode, the reference electrode was SCE and 0.3 M K_2_SO_4_ solution (70 mL) was adopted as the electrolyte. Air (99.99% purity) was bubbled into the anodic compartment with a flow rate of 10 mL min^−1^ in the whole electrochemical process. The linear sweep voltammetry was performed at a rate of 10 mV s^−1^ and the potentiostatic test was tested at a different anodic voltage for 20 h with the electrolyte agitated at a stirring rate of ∼350 rpm. An absorption flask containing 5 mL K_2_SO_4_ solution (0.3 M) was connected to the gas outlet of an anodic half cell to avoid the loss of products due to air bubbling. After the electrooxidation measurements, the components of the mixed solutions in the anodic compartment and absorption flask were both analysed. For comparison, the electrooxidation measurements were also measured with all the testing conditions consistent with aforementioned N_2_ oxidation except that air was replaced by Ar or the external potential was removed.

The electroreduction of ${\rm NO_{3}^{-}}$ to NH_3_ via Strategy II was carried out in a three-electrode configuration with as-prepared Co_3_O_4_ (1 × 1 cm^2^) electrode, SCE and platinum foil as working electrode, reference electrode and counter electrode, respectively; 0.1 M K_2_SO_4_ solution (80 mL) was used as the electrolyte and evenly distributed to the cathode and anode compartment. KNO_3_ (100 g L^−1^) was added into the cathode compartment as a reactant. Prior to the ${\rm NO_{3}^{-}}$ electroreduction test, the cathode electrolyte was purged with Ar (99.99% purity) for 30 min. The linear sweep voltammetry was performed at a rate of 20 mV s^−1^ and the potentiostatic test was conducted at −0.65 V for 3 h at a stirring rate of ∼350 rpm. For comparison, the electroreduction measurements were also conducted with all the testing conditions consistent with the aforementioned ${\rm NO_{3}^{-}}$ reduction except that the Co_3_O_4_ cathode was replaced by the Ti mesh or the Co_3_O_4_ cathode was immersed in electrolyte (0.1 M K_2_SO_4_) without the addition of KNO_3_.

### Ion-concentration detection methods

The electrolytes pre and post test were first diluted to appropriate concentration and then tested using a UV-Vis spectrophotometer to quantify the concentration. The concentrations of nitrate-N, nitrite-N and ammonia-N were estimated by UV-Vis spectrophotometry according to the standard method. The specific approaches are as follows.

#### Determination of nitrate-N

First, a certain amount of electrolyte was taken out of the electrolytic cell and diluted to 5 mL to the detection range. Then, 0.1 mL 1 M HCl and 0.01 mL 0.8 wt% sulfamic acid solution were added to the aforementioned solution. The absorption spectrum was tested using an ultraviolet-visible spectrophotometer and the absorption intensities at wavelengths of 220 and 275 nm were recorded. The final absorbance value was calculated using the equation: *A* = *A*_220nm_ – 2*A*_275nm_. The concentration–absorbance curve was made using a series of standard potassium nitrate solutions and the potassium nitrate crystal was dried at 105–110°C for 2 h in advance.

#### Determination of nitrite-N

A mixture of *p*-aminobenzenesulfonamide (4 g), *N-*(1-Naphthyl)ethylenediamine dihydrochloride (0.2 g), ultrapure water (50 mL) and phosphoric acid (10 mL, ρ = 1.70 g/mL) was used as a color reagent. A certain amount of electrolyte was taken from the electrolytic cell and diluted to 5 mL to the detection range. Next, 0.1 mL color reagent was added into the aforementioned 5-mL solution and mixed to uniformity, and the absorption intensity at a wavelength of 540 nm was recorded after sitting for 20 min. The concentration–absorbance curve was calibrated using a series of standard sodium nitrite solutions.

#### Determination of ammonia-N

Ammonia-N was determined using Nessler's reagent as the color reagent. First, a certain amount of electrolyte was taken from the electrolytic cell and diluted to 5 mL to the detection range. Next, 0.1 mL potassium sodium tartrate solution (ρ = 500 g/L) was added and mixed thoroughly, then 0.1 mL Nessler's reagent was put into the solution. The absorption intensity at a wavelength of 420 nm was recorded after sitting for 20 min. The concentration–absorbance curve was made using a series of standard ammonium chloride solutions and the ammonium chloride crystal was dried at 105°C for 2 h in advance.

The calibration curves of nitrate-N and nitrite-N for the electrooxidation of N_2_ are shown in Supplementary Fig. S1, available as Supplementary Data at *NSR* online (0.3 M K_2_SO_4_ as the background solution). The calibration curves of nitrate-N, nitrite-N and ammonia-N for the electroreduction of ${\rm NO_{3}^{-}}$ are shown in Supplementary Fig. S5, available as Supplementary Data at *NSR* online (ultrapure water as the background solution).

All the results obtained using UV-Vis spectrophotometry were compared with those of ion chromatography (Supplementary Tables S1 and S3, available as Supplementary Data at *NSR* online). The pre-treatment of the cation chromatography measurement of ammonia-N after the electroreduction test was as follows. First, the electrolyte was put into a round flask and then the pH was adjusted to ∼8 by the addition of NaOH. The solution was then heated to ∼100°C and went through condensation until the 90% electrolyte was distilled. The distilled solution was collected using 35 mL ultrapure water and used for the cation chromatography measurement.

### Calculation of the yield, selectivity and Faradaic efficiency

For the N_2_ electrooxidation experiments via Strategy I, the yield of ${\rm NO_{3}^{-}}$ and ${\rm NO_{2}^{-}}$ was calculated using Equation (4) and Equation (5), respectively:

(4) \begin{equation*}{\rm{Yield}}_{{\rm{N}}{{\rm{O}}_{3}^{-}}} = (c_{{\rm{N}}{{\rm{O}}_{3}^{-}}} \times V)/(M_{{\rm{N}}{{\rm{O}}_{3}^{-}}} \times t \times S)\end{equation*}

(5) \begin{equation*}{\rm{Yield}}_{{\rm{N}}{{\rm{O}}_{2}^{-}}} = (c_{{\rm{N}}{{\rm{O}}_{2}^{-}}} \times V)/(M_{{\rm{N}}{{\rm{O}}_{2}^{-}}} \times t \times S).\end{equation*}

The Faradaic efficiency was calculated according to the charge consumed for synthesizing the NO gas and the total charge passed through the electrode using Equation (6):

(6) \begin{eqnarray*} {\rm{Faradaic\,\, efficiency}} &=& (2F \times c_{{\rm{N}}{{\rm{O}}_{3}^{-}}} \times V)\nonumber\\ &&/(M_{{\rm{N}}{{\rm{O}}_{3}^{-}}} \times Q), \end{eqnarray*}

where *c*_NO3_^−^ is the concentration of NO_x_^−^, *V* is the volume of the electrolyte in the anode compartment, *M*_NO3_^−^ is the molar mass of ${\rm NO_{3}^{-}}$, *M*_NO2_^−^ is the molar mass of ${\rm NO_{2}^{-}}$, *t* is the electrolysis time, *S* is the geometric area of the Pt plate, *F* is the Faradaic constant (96 485 C mol^−1^) and *Q* is the total charge passing the electrode. (Note that NO and NO_2_ gas consumed two and four electrons, respectively. We prudently chose NO gas as the product to calculate the Faradaic efficiency.)

For the ${\rm NO_{3}^{-}}$ electroreduction experiments via Strategy II, the yield of NH_3(aq)_ was calculated using Equation (7):

(7) \begin{equation*}{\rm{Yield}}_{{\rm{N}}{{\rm{O}}_{3}^{-}}} = ({c_{{\rm{N}}{{\rm{H}}_3}}} \times V)/({M_{{\rm{N}}{{\rm{H}}_3}}} \times t \times S).\end{equation*}

The conversion of ${\rm NO_{3}^{-}}$ was obtained from Equation (8):

(8) \begin{equation*}{\rm{Conversion}} = \Delta c_{{\rm{N}}{{\rm{O}}_{3}^{-}}} /{c_0} \times 100\% .\end{equation*}

The selectivity of product was acquired by Equation (9):

(9) \begin{equation*}{\rm{Selectivity}} = c/\Delta c_{{\rm{N}}{{\rm{O}}_{3}^{-}}} \times 100\% ,\end{equation*}

where *c*_NH3_ is the concentration of NH_3(aq)_, Δ*c*_NO3_^−^ is the concentration difference of ${\rm NO_{3}^{-}}$ before and after electrolysis, *c*_0_ is the initial concentration of ${\rm NO_{3}^{-}}$, and *c* is the concentration of products, including NH_3(aq)_, ${\rm NO_{2}^{-}}$.

### ^15^N isotope-labeling experiment

The isotope-labeling reactants of ^15^N_2_ (>99 atom% ^15^N) and K^15^NO_3_ (20.3 atom% ^15^N) were purchased from the Shanghai Research Institute of Chemical Industry Co. Ltd. The isotope-labeling concentration of products via both Strategy I and Strategy II was established by isotopic mass spectrometry from the Shanghai Engineering Research Center of Stable Isotope.

For isotope labeling the N_2_ electrooxidation, we carried out the batch experiments using ^15^N_2_ as the feeding gas for five successive times and collected all the electrolytes together after electrolysis. In order to increase the concentration of ^15^${\rm NO_{3}^{-}}$ for isotopic mass spectrometry, the collected electrolytes were first alkalified to pH ∼7 by adding 1 M KOH solution and then concentrated by 10 times via distilling at 70°C. For comparison, ^14^N_2_ with natural-abundance ^15^N (0.36 atom%) was used as a feeding gas to replace ^15^N_2_ with other conditions being consistent.

For isotope labeling the ${\rm NO_{3}^{-}}$ electroreduction, the pH of final electrolyte was adjusted to ∼3 using 1 M HCl solution before isotopic mass spectrometry. For comparison, K^14^NO_3_ with natural-abundance ^15^N (0.36 atom%) was used as the reactant to replace K^15^NO_3_ with the other conditions being consistent.

### Theoretical simulation

Although the XRD pattern of platinum after the electrooxidation process (not shown here) showed no obvious new species, the high oxidation state of PtOH or PtO_x_ might exist in an amorphous state. Considering the complexity of the electrochemical oxidation process, herein, we only adopted Pt as a model for simulation. The theoretical calculations were conducted using density functional theory with the Perdew–Burke–Ernzerbof form of generalized gradient approximation functional [36]. The plane wave energy was cut off at 400 eV. The Vienna *abinitio* simulation package was used [37,38]. The Fermi scheme was used for electron occupancy with an energy smearing of 0.1 eV. The first Brillouin zone was adopted in the Monkhorst−Pack grid [39]. The 3 × 3 × 1 k-point mesh was taken for the surface calculation. The energy of 1.0 × 10^−6 ^eV atom^−1^ and force of 0.01 eV Å^−1^ were set as the convergence criterion for geometry optimization. The spin polarization was considered in all calculations. To accurately describe the van der Waals (vdW) interaction, the non-local van der Waals density functional (vdW-DF) was employed in our work [40,41].

The Pt (200) surface was obtained by cutting the Pt bulk along the {200} direction based on the XRD result (Supplementary Fig. S3, available as Supplementary Data at *NSR* online). The thickness of the surface slab was chosen to be a three-layer slab. In all structural optimization calculations, the bottom atoms were frozen, while the other atoms were allowed to relax. A vacuum layer as large as 15 Å was used along the c direction normal to the surface to avoid periodic interactions. For the nitrogen oxidation reaction, the reaction energies of the elementary reactions were employed to estimate the activity of the catalyst.

The free-energy changes of the nitrogen oxidation were calculated to show the reaction trend. The change in free energy (ΔG) of the per reaction step from the initial state to the final state of the reaction was calculated as:

$$
\begin{equation*}
\Delta G = \Delta E + \Delta ZPE - T\Delta S,
\end{equation*}$$

where Δ*E* is the change in the total reaction energy obtained from the density functional theory (DFT) calculations and Δ*ZPE* is the change in the zero-point energy. *T* is the temperature (298.15 K) and Δ*S* is the change in entropy. The free energy of H^+^ is equal to that of half H_2_ according to the computational hydrogen electrode model proposed by Nørskov [42], while the OH^−^ is estimated by H_2_O → OH^−^ + H^+^. The zero-point energy and the entropies of the adsorbed nitrogen oxidation species were taken from the vibrational frequencies.

## Supplementary Material

nwz019_Supplemental_File
