# Supplementary material for: Electrochemical synthesis of nitric acid from air and ammonia through waste utilization
Source: Natl Sci Rev. 2019 Feb 1;6(4):730–8. doi: 10.1093/nsr/nwz019 (PMC8291439; doi:10.1093/nsr/nwz019)
Supplement: nwz019_Supplemental_File [file nwz019_supplemental_file.docx]

Supplementary Information for

**Electrochemical Synthesis of Nitric Acid from Air and Ammonia through Waste Utilization**

Yuting Wang^1^, Yifu Yu^1^*, Ranran Jia^1^, Chao Zhang^1^ & Bin Zhang^1,2^*

^1^Department of Chemistry, Institute of Molecular Plus, School of Science, Tianjin University, Tianjin 300072, China

^2^Collaborative Innovation Center of Chemical Science and Engineering, Tianjin 300072, China

*e-mail: bzhang@tju.edu.cn; yyu@tju.edu.cn


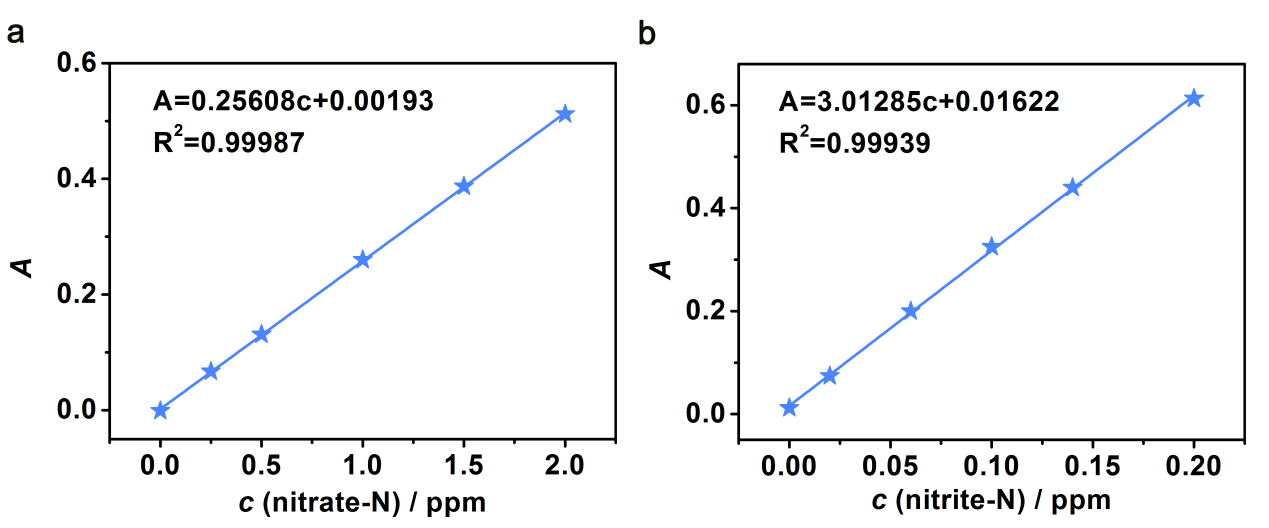


**Supplementary Figure 1.** The UV-Vis absorption spectra and the corresponding calibration curves of **a**) nitrate-N, **b**) nitrite-N for N_2_ electrooxidation measurements by using 0.3 M K_2_SO_4_ as background solution.


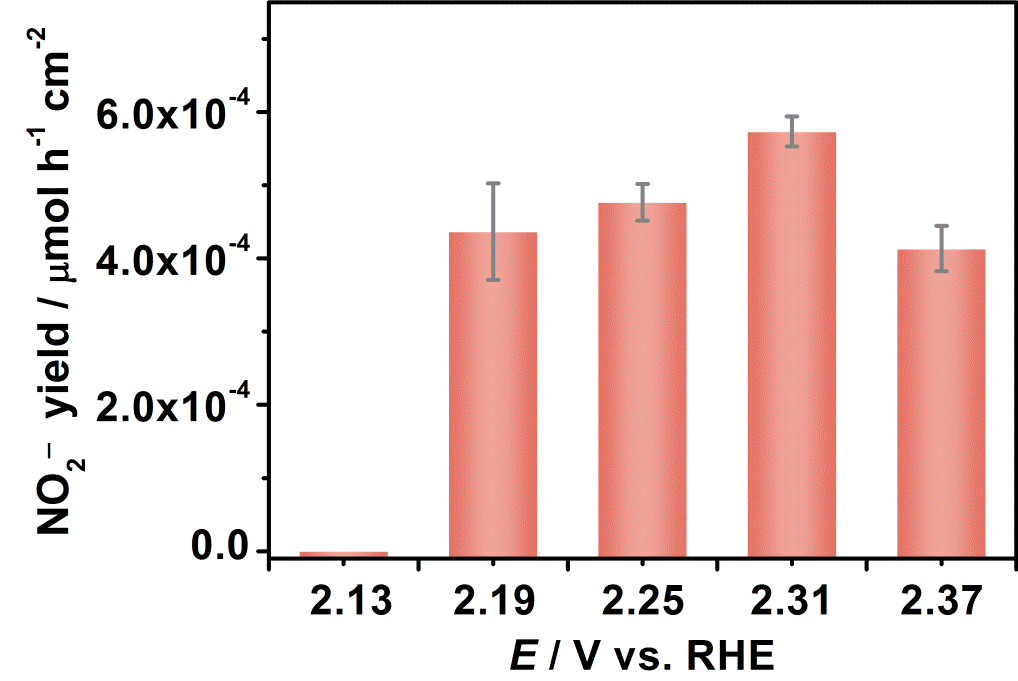


**Supplementary Figure 2.** NO_2_^-^ yield at different potentials in N_2_ electrooxidation reaction. NO_2_^-^ can be found in electrolyte after electrooxidation tests, confirming the successful oxidation of N_2_.


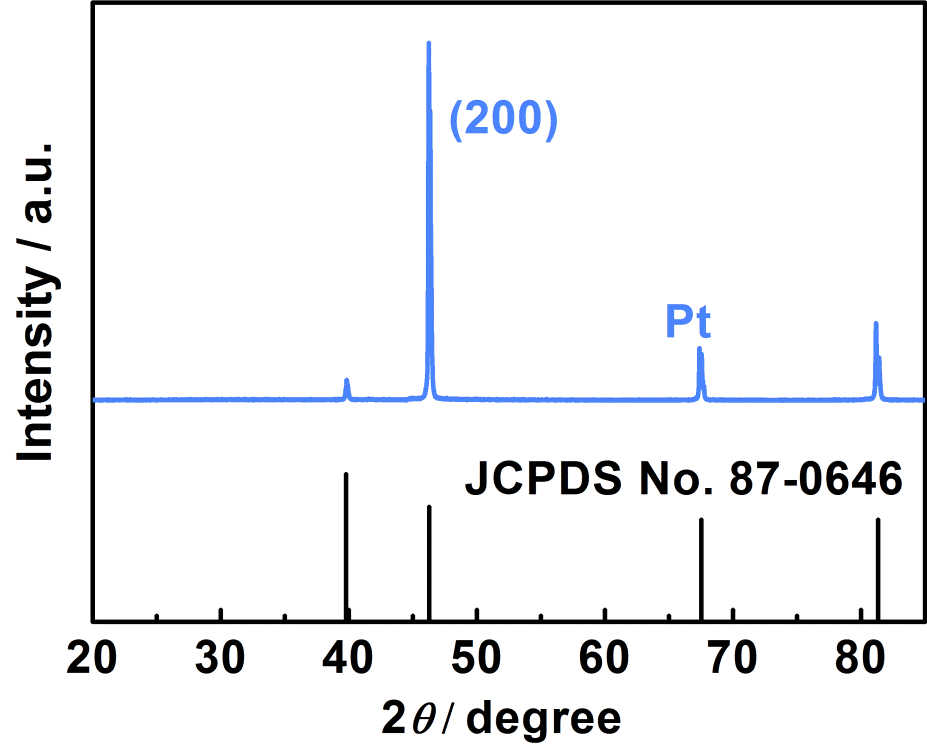


**Supplementary Figure 3.** XRD pattern of Pt plate. The Pt (200) plane is the strongest peak and thus chosen as model for the subsequent theoretical calculation. (JCDPS: the Joint Committee on Powder Diffraction Standards).


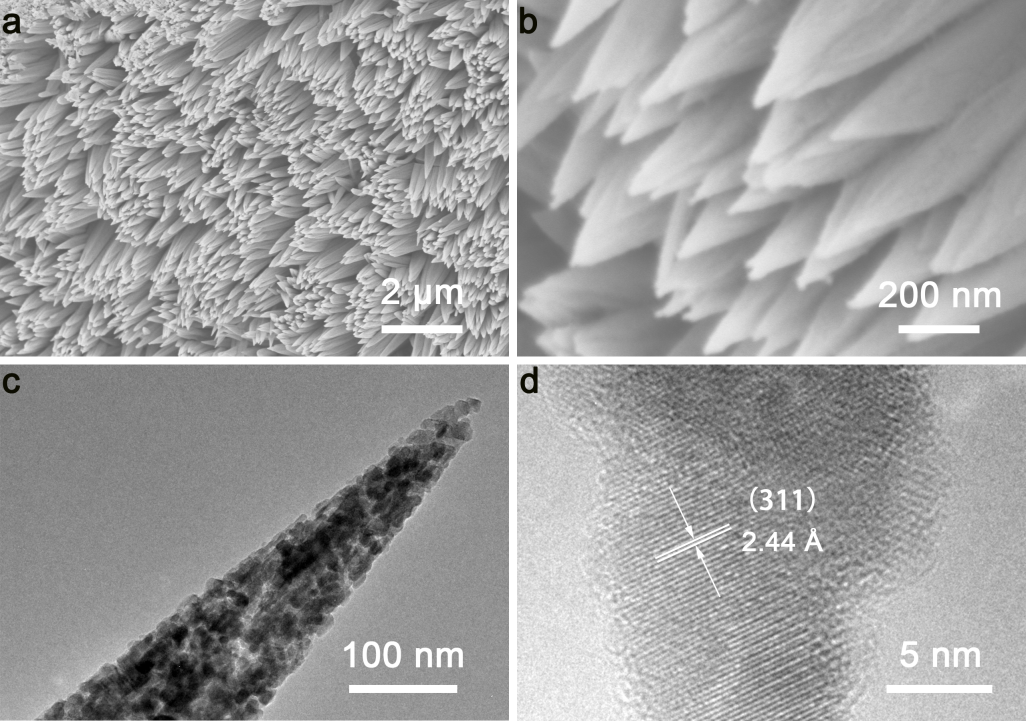


**Supplementary Figure 4. a,b**) SEM images, **c**) TEM image, **d**) HRTEM image of Co_3_O_4_ electrocatalyst. These results indicated that Co_3_O_4_ nanorod arrays supported on Ti mesh was successfully prepared.


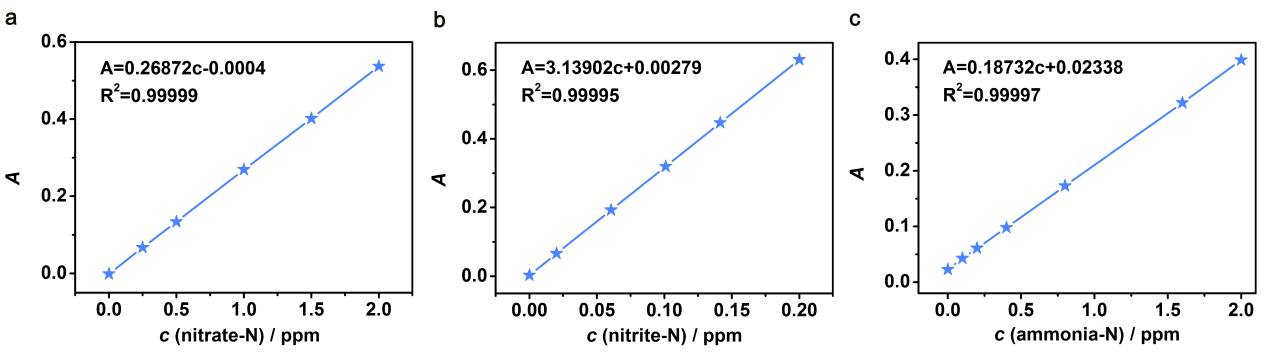


**Supplementary Figure 5.** The UV-Vis absorption spectra and the corresponding calibration curves of **a**) nitrate-N, **b**) nitrite-N, **c**) ammonia-N for NO_3_^‑^ electroreduction measurements by using ultrapure water as background solution.


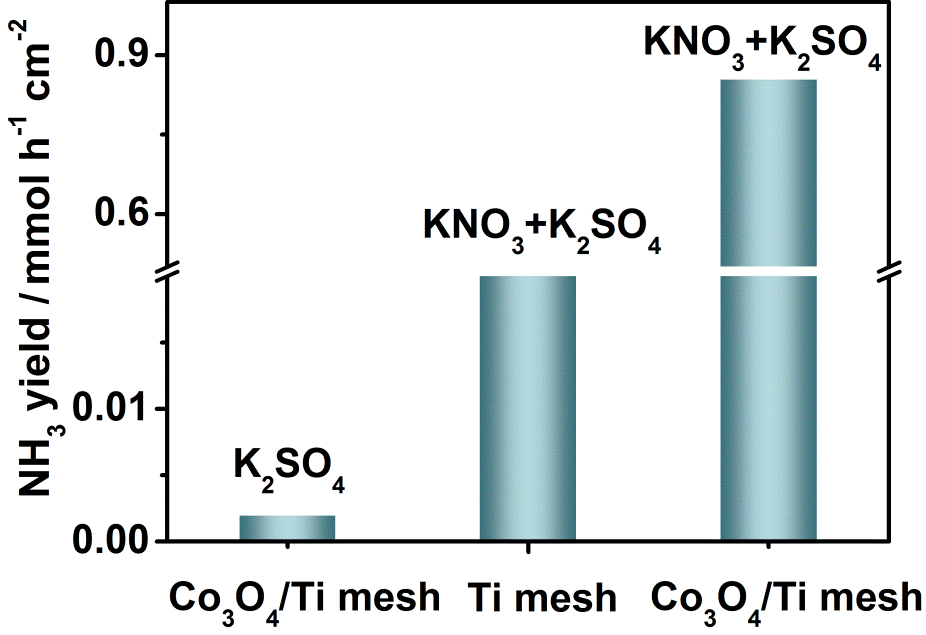


**Supplementary Figure 6.** NH_3(aq)_ yield after NO_3_^-^ reduction under different conditions at -0.65 V for 3 h. Co_3_O_4_/Ti mesh in the K_2_SO_4_ electrolyte without the presence of KNO_3_ generated trace NH_3(aq)_ (0.002 mmol h^-1^ cm^-2^), which may arise from residual NO_3_^-^ in K_2_SO_4_ chemicals. Compared with the Co_3_O_4_/Ti mesh, Ti mesh generated much fewer NH_3(aq)_ (0.029 mmol h^-1^ cm^-2^) in the K_2_SO_4_ electrolyte with the presence of KNO_3_, demonstrating the high activity of Co_3_O_4_ for electroreduction of NO_3_^-^.


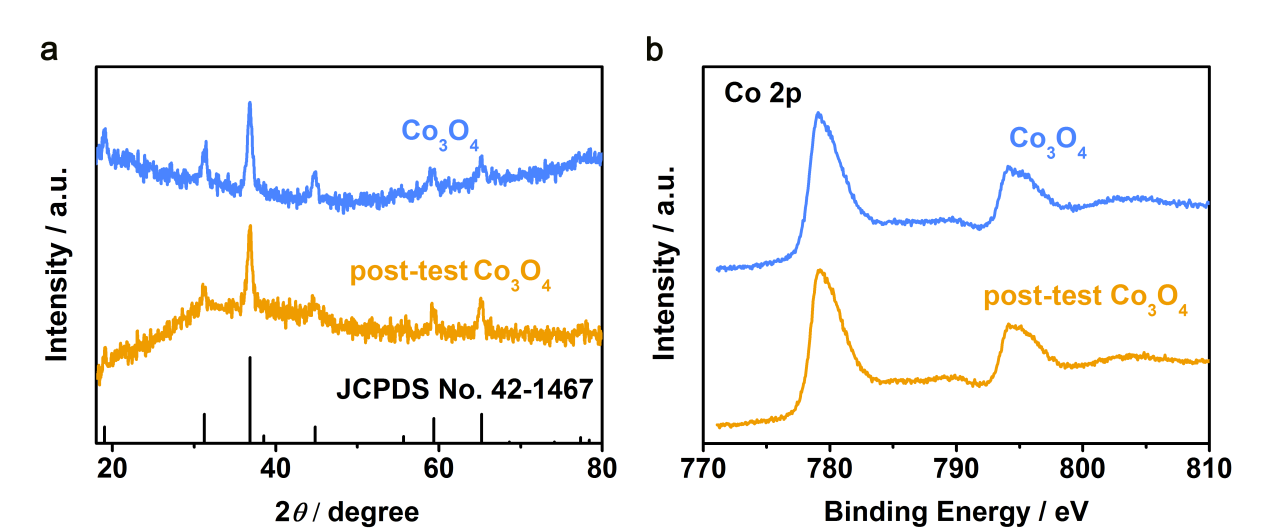


**Supplementary Figure 7.** **a**) XRD pattern of the Co_3_O_4_ electrode before and after electrochemical tests, **b**) XPS spectra of Co 2p peaks in Co_3_O_4_ electrode before and after electrochemical tests. These results demonstrated the high durability of Co_3_O_4_ electrode for NO_3_^-^ electroreduction.

**Supplementary Table 1.** Comparison of the quantitative approach between anion chromatography (AC) and UV-Vis spectrophotometry for NO_3_^-^ and NO_2_^-^ analysis in standard solution.

| Anion ions | AC  (deionized water) | UV-Vis  (deionized water) | UV-Vis  (0.3 M K_2_SO_4_) | Theoretical concentration |
| --- | --- | --- | --- | --- |
| NO_3_^-^ | 5.0055 ppm | 5.6900 ppm | 5.6563 ppm | 5.5357 ppm |
| NO_2_^-^ | 5.4403 ppm | 5.1330 ppm | 5.1360 ppm | 5.1339 ppm |

Anion chromatography and UV-Vis spectrophotometry were performed to quantify the concentration of NO_3_^-^ and NO_2_^-^ of standard solution. Ion chromatography can’t be applied to salt solution with high concentration. So, the concentrations of NO_3_^-^ and NO_2_^-^ in both deionized water and 0.3 M K_2_SO_4_ were quantified by UV-Vis spectrophotometry, while anion chromatography was used to analyze the concentration of NO_3_^-^ and NO_2_^-^ in deionized water. All the data are similar to the theoretical values, confirming the feasibility of ultraviolet-visible spectrophotometry and anion chromatography for detecting the concentration of NO_3_^-^ and NO_2_^-^.

**Supplementary Table 2.** Control experiments for N_2_ electrooxidation reaction.

| Experiment condition | NO_3_^-^ yield | NO_2_^-^ yield |
| --- | --- | --- |
| Pt plate in Ar-saturated 0.3 M K_2_SO_4_  electrolyte performed at +2.19 V for 20 h | undetected | undetected |
| Pt plate in air-saturated 0.3 M K_2_SO_4_ electrolyte performed without external potential for 20 h | undetected | undetected |

There are no oxidative products in both comparison tests, further confirming that N_2_ in air was electro-catalyzed into oxidative products over Pt plate via Strategy I.

**Supplementary Table 3.** Comparison of the quantitative approach between cation chromatography (CC) and UV-Vis absorption spectra for NH_3(aq)_ analysis after electroreduction test.

|  | CC (deionized water) | UV-Vis (post-test electrolyte) |
| --- | --- | --- |
| Ammonium ion | 1.7544 ppm | 1.7537 ppm |

Cation chromatography and UV-Vis spectrophotometry were performed to quantify the concentration of NH_3(aq)_ in post-test electrolyte. Ion chromatography can’t be applied to salt solution with high concentration. To avoid the interference of K^+^, the NH_3(aq)_ in post-test electrolyte was first distilled at 100 ℃ and then absorbed by deionized water. Finally, the purified NH_3(aq)_ in deionized water was quantified by cation chromatography, and the NH_3(aq)_ in post-test electrolyte without distilling treatment was directly quantified by UV-Vis spectrophotometry. Both values are quite similar, confirming the feasibility of UV-Vis spectrophotometry for detecting the concentration of NH_3(aq)_ in post-test electrolyte.
